# Structure-dependent genotoxic potencies of selected pyrrolizidine alkaloids in metabolically competent HepG2 cells

**DOI:** 10.1007/s00204-020-02895-z

**Published:** 2020-09-10

**Authors:** Lukas Rutz, Lan Gao, Jan-Heiner Küpper, Dieter Schrenk

**Affiliations:** 1 Food Chemistry and Toxicology, University of Kaiserslautern, Kaiserslautern, Germany; 2 Institute of Biotechnology, Brandenburg University of Technology Cottbus-Senftenberg, Senftenberg, Germany

**Keywords:** Genotoxicity, Liver cells, Micronuclei, Mutagenicity, Pyrrolizidine alkaloids, Relative potencies

## Abstract

1,2-unsaturated pyrrolizidine alkaloids (PAs) are natural plant constituents comprising more than 600 different structures. A major source of human exposure is thought to be cross-contamination of food, feed and phytomedicines with PA plants. In humans, laboratory and farm animals, certain PAs exert pronounced liver toxicity and can induce malignant liver tumors in rodents. Here, we investigated the cytotoxicity and genotoxicity of eleven PAs belonging to different structural classes. Although all PAs were negative in the fluctuation Ames test in *Salmonella*, they were cytotoxic and induced micronuclei in human HepG2 hepatoblastoma cells over-expressing human cytochrome P450 3A4. Lasiocarpine and cyclic diesters except monocrotaline were the most potent congeners both in cytotoxicity and micronucleus assays with concentrations below 3 μM inducing a doubling in micronuclei counts. Other open di-esters and all monoesters exhibited weaker or much weaker geno- and cytotoxicity. The findings were in agreement with recently suggested interim Relative Potency (iREP) factors with the exceptions of europine and monocrotaline. A more detailed micronuclei analysis at low concentrations of lasiocarpine, retrorsine or senecionine indicated that pronounced hypolinearity of the concentration–response curves was evident for retrorsine and senecionine but not for lasiocarpine. Our findings show that the genotoxic and cytotoxic potencies of PAs in a human hepatic cell line vary in a structure-dependent manner. Both the low potency of monoesters and the shape of prototype concentration–response relationships warrant a substance- and structure-specific approach in the risk assessment of PAs.

**Electronic supplementary material:**

The online version of this article (10.1007/s00204-020-02895-z) contains supplementary material, which is available to authorized users.

## Introduction

A large number of plants contain 1,2-unsaturated pyrrolizidine alkaloids (PAs) known for their toxic properties. Among those are plants traditionally used as food, in particular herbal teas (Bodie et al. 2014), feed or herbal medicine (Schulz et al. 2015). In addition, PA-containing plants may contaminate non-PA plants and products thereof with serious consequences for their marketing and use (Mulder et al. 2018; Steinhoff 2019). Exposure to certain PAs at critical dose levels can lead to dramatic signs of acute toxicity such as body weight loss, liver failure and death in experimental, wild and farm animals (Williams and Molyneux 1987; Stegelmeier et al. 1999; Woolford et al. 2014). Intoxications of humans have also been described after consumption of PA plants or teas made from these (Stewart and Steenkamp 2001). Furthermore, chronic exposure can result in liver failure characterized by a hepato-venous occlusive disease (HVOC) with destruction of venous endothelial cells in the liver. Severe consequences include internal bleeding, portal hypertension and cirrhosis (Stegelmeier et al. 1999). Probably on the basis of such damage, malignant tumors of the endothelia (sarcoma) or hepatocytes (carcinoma) may develop and were observed in laboratory rodents chronically treated with PAs (Fu et al. 2004).

Certain 1,2-unsaturated PA congeners exert genotoxic properties, i.e., they can form DNA adducts, elicit mutations in the Drosophila wing test, and are genotoxic in a variety of in vitro assays. Recent studies have revealed that some selected PAs are weakly or unequivocally mutagenic in the Ames test (Rubiolo et al. 1992; Wehner et al. 1979; Yamanaka et al. 1979), whereas they were able to induce micronuclei formation in mammalian cells (Allemang et al. 2018).

It is widely accepted that DNA binding and genotoxicity depend on the formation of reactive PA metabolite(s) mainly formed in the liver catalyzed by cytochrome P450 (CYP) mono-oxygenases (Li et al. 2011). Upon oxidation of the 1,2-unsaturated necine base, e.g., the retronecine ring of the PA molecule, a reactive, electrophilic dehydroretronecine, either as intact ester or after ester hydrolysis, is formed which binds covalently to DNA (Fu et al. 2004). It is also discussed that secondary metabolites formed from binding of dehydroretronecine to nucleophiles may still retain some reactivity and cause further damage (Xia et al. 2015). These findings support the notion that certain PAs act as genotoxic carcinogens and illustrate the need for an adapted toxicological risk assessment taking into account the risk from chronic exposure to low dose levels (Schrenk et al. 2020).

Since PAs are always synthesized and occur in plants as mixtures of various congeners, the risk arising from simultaneous exposure to different congeners has to be assessed. The toxic and genotoxic potencies of different congeners have been shown, however, to vary by more than two orders of magnitude (Merz and Schrenk 2016). Thus, the current approach to analyze a certain set of PAs in a sample and attribute all of them with the same potency as the most toxic congeners (lasiocarpine or riddelliine) is a clear over-estimation of the risk. Recently, we have tried to generate a suggestion for potency factors for prototypic PAs representing certain structural classes (Merz and Schrenk 2016). These data indicate that cyclic diesters and some open-chained diesters are the most toxic ones, followed by other open-chained diesters and monoesters. A classification of structures based on limited datasets from rodents, Drosophila and in vitro studies, thus, allowed the assignment of interim relative potency (iREP) factors ranging from 1.0 (for the most toxic PAs) to 0.01 (for the least toxic congeners). In was argued, however, that the basis for this classification is uncertain and needs further refinement before in can be taken into account in quantitative risk assessment (EFSA 2017).

In recent publications, good agreement was found in various in vitro test systems for cytotoxicity (Gao et al. 2019), micronucleus formation (Allemang et al. 2018), DNA adduct formation (Lester et al. 2019), and indirect markers for genotoxicity (Louisse et al. 2019) between toxic potencies in the assay and iREP factors for various PAs. For some congeners, however, especially for echimidine and monocrotaline, substantial deviations were found.

In this study, we applied systems for in vitro mutagenicity (fluctuation Ames test in *Salmonella typhimurium*) and clastogenicity/aneugenicity (micronucleus assay in mammalian cells) to a set of eleven frequently occurring PAs. We also measured cytotoxicity both in bacteria and in mammalian cells to avoid false-negative genotoxicity findings. We used two different *Salmonella* strains, TA 98 (detection of frameshift mutations) and TA 100 (detection of base pair substitutions), as well as a stably transfected human HepG2 hepatoblastoma cell line expressing human CYP3A4, one of the major enzymes catalyzing the metabolic activation of PAs (Ruan et al. 2014). The major aims of this study were the further refinement of the concept of relative toxic potency of PA congeners as well as an investigation of the concentration–response characteristics at very low, more relevant PA concentrations.

## Materials and methods

### Chemicals, media and cells

Dulbecco’s modified Eagle’s Medium–High Glucose (DMEM-HG), Pen/Strep solution, and fetal calf serum (FCS) were from Life Technologies (Paisley, UK), blasticidin S hydrochloride from Carl Roth (Karlsruhe, Germany), dicumarol, methyl methane sulfonate (MMS), saponin, trypan blue solution and resorufin benzyl ether from Sigma-Aldrich (Merck, Darmstadt, Germany), 4′,6-diamidino-2-phenylindole (DAPI) from AppliChem (Darmstadt, Germany). All PAs with the exception of monocrotaline were from Phytolab (Vestenbergsgreuth, Germany). All other chemicals were of the highest purity commercially available.

Monocrotaline was isolated from dried *Crotalaria mirabilis* seeds obtained from Germiterra Ltda. (Barreiras, Brazil). The seeds were ground and the fine powder (3 × 30 g) was extracted with methanol (3 × 300 ml) for 2 × 24 h each in a Soxhlet apparatus. After 24 h, the solution was replaced by fresh methanol. The combined extracts were reduced by evaporation to a volume of approximately 100 ml and stored for 8 h at 4 °C. Precipitated allantoin was removed by filtration and a dark, oily residue was obtained after evaporation of the solvent. The residue was dissolved in 150 ml 5% aqueous HCl and extracted three times with 120 ml chloroform. The aqueous phase was treated with 25% aqueous NH_4_OH until a pH value of 12 was achieved. Then, the solution was extracted three times with 120 ml chloroform and the combined organic phases were evaporated to dryness. The white, yellowish residue was monocrotaline as revealed by elementary analysis, ^1^H-NMR and ^13^C-NMR. The latter techniques and TLC separation (visualized with *o*-chloroanil in toluene) revealed a purity of about 97%. In addition, all PAs were analyzed for impurities by LC/MS/MS analysis as described previously (Geburek et al. 2020). None of the PAs was contaminated by other congeners above their LODs except for heliotrine containing 0.5% heliosupine.

*Salmonella typhimurium* strains TA98 and TA100 were from Molecular Toxicology Inc. (Moltox, Boone, USA). HepG2 cells were from DSMZ, Heidelberg, Germany. HepG2 CYP3A4 cells (clone C9) overexpressing human CYP3A4 were genetically engineered and propagated as previously described (Herzog et al. 2015).

### Fluctuation Ames test

Animal experiments were performed according to National Animal Welfare Regulations after authorization by the local authorities (Struktur- und Genehmigungsbehörde Rheinland-Pfalz, Koblenz, Germany). We used Sprague–Dawley male rats weighing approximately 200 g. To induce rat liver enzymes, we administered 500 mg/kg b.w. Aroclor 1254 via i.p. injection five days before killing from a stock solution of 200 mg/ml diluted in corn oil. The rats were given drinking water and commercial lab chow ad libitum. All steps of preparing the rat liver S9 fraction were carried out at 4 °C using cold and sterile solutions. The livers were washed in 0.15 M KCl and transferred to a beaker containing 3 ml KCl (0.15 M) per g liver. Homogenization was performed with a Potter–Elvehjem apparatus. The homogenate was centrifuged for 10 min at 9000 g and the supernatant (S9 fraction) was stored at − 80 °C.

The Ames fluctuation assay was performed as described in ISO 11350 (2012). Mutagenicity of the test compounds was analyzed in different strains, TA98 and TA100, with and without exogenous metabolic activation by rat liver S9 fraction. The overnight culture of bacteria was grown in nutrient broth medium (Oxoid Ltd., Basingstoke, UK) containing ampicillin (50 µg/ml) in an incubation shaker for 5 h at 37 °C and 125 rpm. The bacterial density was measured with a spectrophotometer at a wavelength of 595 nm. Bacteria were adjusted with exposure medium to reach an optical FAU (formazine attenuation unit) value which differs from strain to strain (TA98: 180, TA100: 45) (ISO 11350, 2012). Per test compound (dissolved in DMSO or in a DMSO/acetonitrile mixture, see below) 10 µl were placed in a 24-well plate, 490 µl bacteria were added and, in case of metabolic activation, 17 µl S9 mix (3%). The plates were incubated at 37 °C and 125 rpm for 100 min. Thereafter, 2.5 ml histidine-deficient indicator medium was added to each well and then transferred into a 384-well microplate. 48 wells were filled each with 50 µl per concentration. The 384-well plates were incubated at 37 °C for 48 h. The reversion from his- to his + in the mutagenic samples was detected by the color change of the pH indicator from purple to yellow. DMSO was used as negative control to detect spontaneous reversions (max. 10 positive wells/48 wells). Positive controls were used to examine the efficiency of the test system. The percentage of positive (yellow) wells in the positive control has to be more than 52% (25 wells/48 wells). All assays were carried out in triplicates. To indicate mutagenicity, the samples had to show a concentration-dependent statistically significant increase of positive wells above background.

### Cell culture and cytotoxicity assay

For standard cytotoxicity testing, HepG2 cells and HepG2 CYP3A4 cells were seeded on 48-well plates (65,000 cells per well). Medium was replaced by fresh medium after 24 h and cultures were incubated with 0.1% DMSO (solvent control), 0.1% saponin (positive control) or with PA. For testing of PAs, cells were incubated with various concentrations of echimidine, europine, heliotrine, indicine, lasiocarpine, lycopsamine, monocrotaline or riddelliine, dissolved in DMSO, or retrorsine, senecionine or seneciphylline dissolved in a 1:1 mixture of DMSO and acetonitrile, for additional 24 or 48 h. The concentrations used were limited by the solubility of the PAs in the medium. The concentration ranges are shown in the respective figures (see below) and were selected according to previous experience with cytotoxicity testing of different PAs in mammalian cell culture (Gao et al. 2020).

Then, medium was removed and cells were analyzed for cytotoxicity using the resazurin reduction assay (Berg et al. 2015).

To obtain information on cytotoxic effects over a longer incubation time (about 1.5 cell cycles) used in the micronucleus assay, cells were also maintained over additional 72 h (recovery) without PA, medium was removed and cells were analyzed for resazurin reduction.

### 7-Benzoxyresorufin-O-dealkylase (BROD) assay

7-Benzyloxyresorufin is used as a model substrate for various human CYP enzymes including CYP3A4 (Alexander et al. 1999). The HepG2 or HepG2 CYP3A4 cells were cultured as described above, the monolayers were rinsed twice with PBS (phosphate-buffered saline, pH 7.4) to remove detached cells. The measurement was started by addition of 1 ml/well assay mixture, i.e., 1 ml PBS containing MgCl_2_ (5 mM), dicumarol (10 µM) and 7-benzyloxyresorufin (5 µM). The amount of resorufin formed by *O*-dealkylation of 7-benzoxyresorufin was determined every 90 s up to 30 min fluorometrically (λ_Ex_ = 544 nm, λ_Em_ = 590 nm) with a Thermo Scientific Fluoroskan Ascent FL. After the measurement, the plate was washed with PBS again and then frozen for protein determination. Resorufin standards (0.5–500 nM) were prepared by diluting a PBS solution. BROD activities were normalized to protein content that was determined with a commercially available Pierce bicinchoninic acid protein assay kit (Pierce, Rockford, USA).

### Micronucleus assay

For the micronucleus test, we followed published standard procedures (Lehmann et al. 2006) with some minor modifications. HepG2 CYP3A4 cells (7 x 10^5^ cells) were seeded on 60 mm Petri dishes. After 24 h in culture (4 ml DMEM low glucose, 10% FCS, 65 µM blasticidin S hydrochloride per dish), medium was removed, test compounds were added in DMSO together with fresh DMEM high glucose, with 10% FCS and 65 µM blasticidin S hydrochlorideand incubated for 24 h. Then, the medium was replaced by fresh medium and the cells were incubated for additional 72 h. Then, the medium was removed, the cell layers were rinsed with 2 ml PBS and trypsinized with trypsin–EDTA solution (0.01%, 300 µl). Then, the cell pellets were rinsed again with PBS, 3 ml ethanol were added each and the pellets were placed in the freezer at − 20 °C for 30 min. After fixation, the liquid supernatant was removed, 750 µl DAPI staining solution was added in the dark, and the flasks were kept at − 20 °C for 10 min. Then, the cultures were inspected with a light microscope equipped with a UV lamp and a 631.25 immersion oil objective. 1000 nuclei per sample were inspected for the occurrence of micronuclei. Micronuclei showed a diameter of 1/16 to 1/3 of the main nucleus, were clearly separated from the main nucleus without overlap, and showed a strong, typical chromatin staining. The number of micronuclei was expressed as micronuclei-containing cells per 1000 cells. Controls were treated with DMSO (vehicle) only (negative control) or with methyl methane sulfonate (positive control).

### Statistical analysis and modeling

**Fig. 1:**
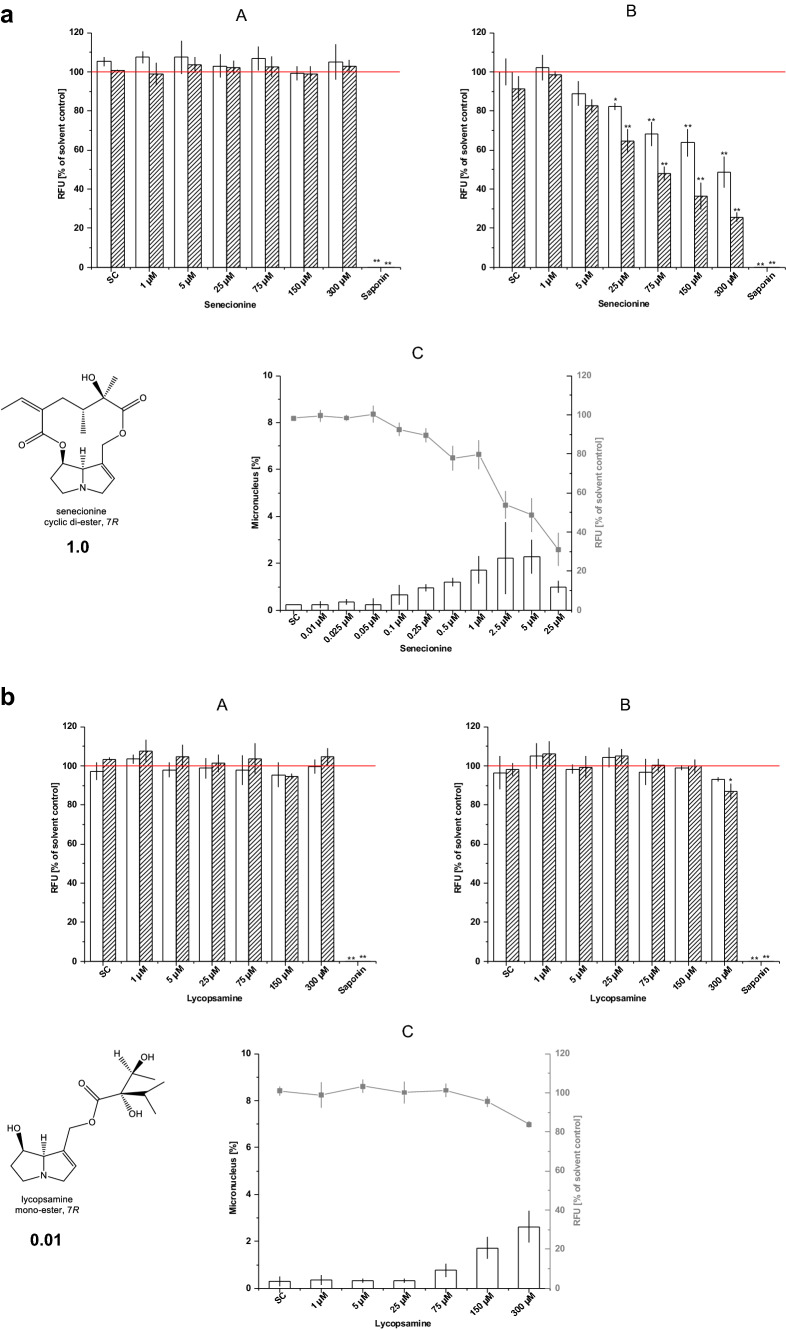
**a** Cytotoxicity and micronuclei formation in HepG2 cells (upper left), HepG2-CYP3A4 cells (upper right) and micronuclei levels (lower, bars) in HepG2-CYP3A4 cells treated with  senecionine. The cytotoxicity in HepG2-CYP3A4 cells under the conditions of the micronuclei assay (with recovery) is also shown (-■-). Data represent mean ± S.D. from *n* = 3 independent experiments. **b** Cytotoxicity and micronuclei formation in HepG2 cells (upper left), HepG2-CYP3A4 cells (upper right) and micronuclei levels (lower, bars) in HepG2-CYP3A4 cells treated with  lycopsamine. The cytotoxicity in HepG2-CYP3A4 cells under the conditions of the micronuclei assay (with recovery) is also shown (-■-). Data represent mean ± S.D. from *n* = 3 independent experiments

Data in Fig. 1 are expressed as mean ± S.D. The data of the test compounds were analyzed with Dunnett’s multiple comparison test for significant (*p* ≤ 0.05) or highly significant (*p* ≤ 0.01) differences from the negative control.

**Fig. 2:**
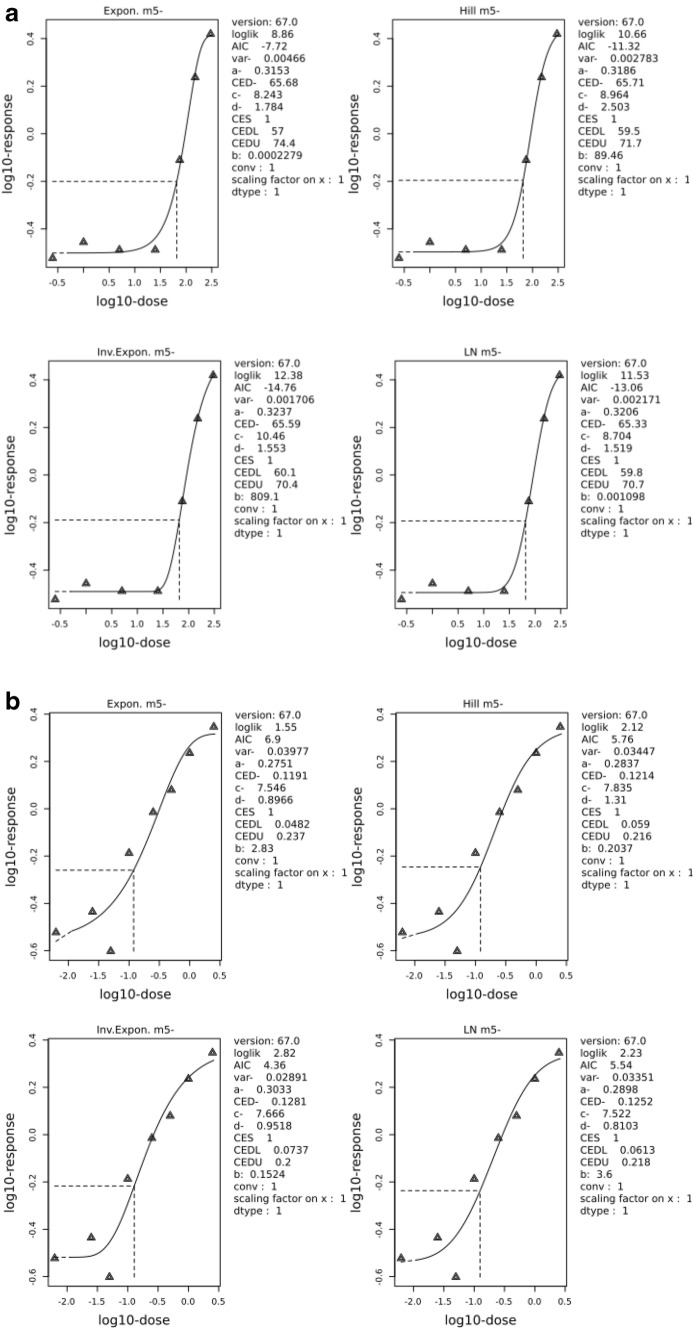
**a** Benchmark-Dose modeling of micronuclei data in HepG2-CYP3A4 cells treated with various concentrations of lycospamine. **b** Benchmark-Dose modeling of micronuclei data in HepG2-CYP3A4 cells treated with various concentrations of senecionine. The panel shows the four best fits using PROAST (EFSA) software and the concentration estimate for a Benchmark effect of doubling of micronuclei counts (for details see ‘Materials and Methods’)

**Fig. 3:**
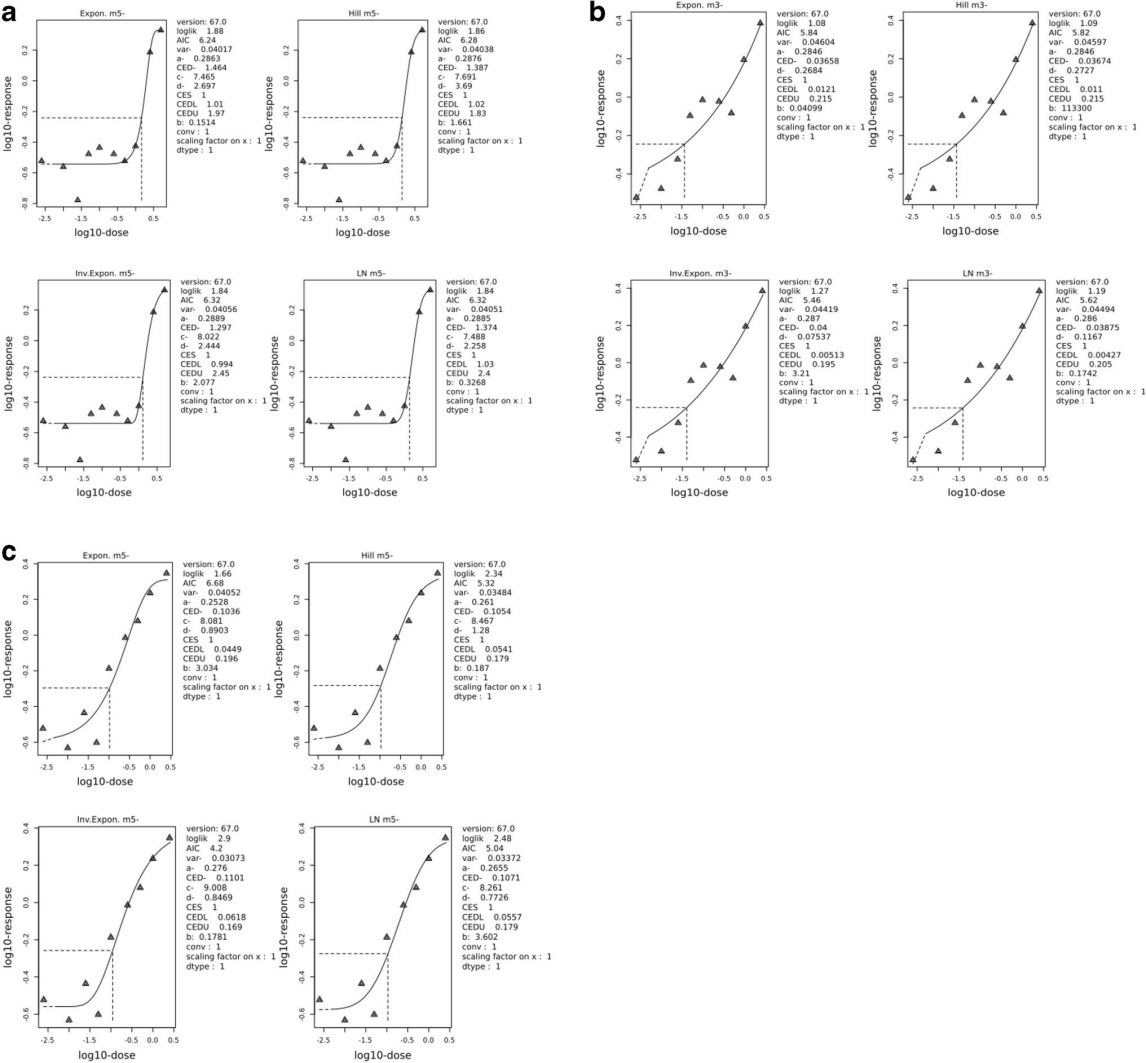
**a** Benchmark-Dose modeling of micronuclei data in HepG2-CYP3A4 cells treated with various concentrations of retrorsine, with a special focus on the low concentration range below 1 μM. **b** Benchmark-Dose modeling of micronuclei data in HepG2-CYP3A4 cells treated with various concentrations of lasiocarpine, with a special focus on the low concentration range below 1 μM. **c** Benchmark-Dose modeling of micronuclei data in HepG2-CYP3A4 cells treated with various concentrations of senecionine, with a special focus on the low concentration range below 1 μM. The panels show the four best fits using PROAST (EFSA) software and the concentration estimates for a Benchmark effect of doubling of micronuclei counts (for details see ‘Materials and Methods’)

For modeling of concentration–response relationships (Figs. 2 and 3), we applied the benchmark calculation mode recommended by RIVM and EFSA (EFSA 2019). This software (PROAST) calculates the best fit of six different algorithms widely used in dose–response modeling. The best four out of these fits are analyzed further and weighted according to their quality of fitting. A doubling of micronuclei counts was used as a benchmark response.

## Results

### Ames fluctuation test

In a first approach, we incubated eleven PAs belonging to different structural classes of 1,2-unsaturated congeners in the fluctuation Ames test. It was found that up to a concentration causing a more than 50% loss in bacterial viability, measured as resazurin reduction, all results were negative (see Supplementary Material). For some PAs, the maximum concentration used was 300 µM; while, others such as senecionine were more toxic to the bacteria and were, thus, applied at lower maximum concentrations (data not shown). The addition of rat liver S9-mix had no effect on the outcome of the fluctuation assay.

### BROD activity

For further experiments, we used the human HepG2 C9 cell line over-expressing the CYP3A4 gene. We determined a mean benzoxyresorufin-*O*-dealkylase (BROD) activity in homogenates of 0.4 pmol/mg protein per min, while the mean activity in non-transformed HepG2 cells was 0.09 pmol/mg protein per min. For comparison, the mean BROD activity in rat hepatocytes was 22.5 ± 11.9 after three h and 1.2 ± 0.3 (in pmol/mg protein per min) after 24 h in culture (unpublished data from experiments in Gao et al. 2020).

### Cytotoxicity

Next, we incubated naïve HepG2 cells and the clone HepG2 C9 (CYP3A4) cells with various concentrations of eleven selected PAs (echimidine, europine, heliotrine, indicine, lasiocarpine, lycopsamine, monocrotaline, retrorsine, riddelliine, senecionine, and seneciphylline) and measured the cytotoxicity in the resazurin reduction assay after incubation over 24 and 48 h. Findings were confirmed by microscopical inspection of the cultures. In a different protocol, micronuclei counts were determined after a PA treatment over 72 h (see below). The longer treatment was required because of the special requirements for the formation of micronuclei, i.e., cytotoxicity testing over 1.5–2 cell cycles as recommended by OECD (2016). In Fig. 1, the effects of two ‘prototype’ PAs, i.e., the cyclic diester senecionine and the monoester lycopsamine are shown. Senecionine was non-toxic in naïve HepG2 cells up to a concentration of 300 µM, while a significant cytotoxicity was seen at 25 µM and above in HepG2 C9 cells expressing the human CYP3A4 gene (Fig. 1a). Treatment over 48 h exerted a somewhat higher cytotoxicity than the 24 h treatment. In incubations over 72 h, a continuous decline in viability being below 20% at 25 µM was found.

The monoester lycopsamine was also non-toxic in naïve HepG2 cells, and almost non-toxic in HepG2 C9 cells with a significant but minor decrease in viability after 48 h at the highest concentration tested (300 µM). Cytotoxicity was also determined over 72 h showing a very minor decline in viability which remained above 80% at all concentrations tested.

Cytotoxicity data obtained for all eleven PAs after treatment over 48 h were modeled using sigmoidal curve fitting and half-maximally cytotoxic concentrations were calculated (Table 1). With some of the PAs, a substantial cytotoxicity was measured. It was found that the cytotoxicity strongly depended on the individual PA congener. With some congeners, major cytotoxicity was found in the lower micromolar range, while others did not reach EC_50_ concentrations even when over 300 μM was added, a finding also seen in rat hepatocytes in primary culture (Table 1). In the non-transfected HepG2 cells tested, none of the PAs exhibited significant cytotoxicity up to a concentration of 300 μM (data not shown). When compared to previously published EC_50_ values of cytotoxicity in PH (Gao et al. 2020), HepG2 C9 cells turned out to be less sensitive towards most congeners tested, while heliotrine was about equally cytotoxic in both cell types. Congeners with low (EC_50_ > 300 µM) or relatively low (> 100 µM) cytotoxicity in PH were also weakly cytotoxic in HepG2 C9 cells. A comparison with iREP factors revealed a marked overestimation of the cytotoxicity of monocrotaline within the most toxic group assigned with an iREP factor of 1.0, and a marked overestimation of the cytotoxicity of europine (iREP factor 0.3).

**Table 1:** Cytotoxicity and micronuclei formation in HepG2-CYP3A4 cells treated with selected PA: Half-maximally effective concentrations (EC50s) of cytotoxicity in HepG2-CYP3A4 cells treated over 48 h with selected pyrrolizidine alkaloids (PAs); Upper Bound and Lower Bound levels of concentrations causing a doubling of micronuclei counts were calculated using PROAST/EFSA software for Benchmark-Dose calculation

PA (structural features)	Cytotoxicity HepG2-CYP3A4 EC50 (μM)	Cytotoxicity PH EC50 (μM), 48 h, treated 3 h after seeding	Benchmark concentration (μM) of doubling of micronuclei counts in HepG2-CYP3A4 cells (Lower Bound)	Benchmark concentration (μM) of doubling of micronuclei counts in HepG2-CYP3A4 cells (Upper Bound)	iREP
Lasiocarpine (open, di, *7S*)	10 ± 1	4 ± 1	0.01	0.49	1.0
Monocrotaline (cyclic, di,*7R*)	> 300	> 300	23.7	153	1.0
Retrorsine (cyclic, di, *7R*)	73 ± 12	19 ± 2	1.26	1.90	1.0
Riddelliine (cyclic, di, *7R*)	97 ± 13	8 ± 1	1.29	2.29	1.0
Senecionine (cyclic, di, *7R*)	67 ± 8	8 ± 1	0.05	0.24	1.0
Seneciphylline (cyclic, di,*7R*)	73 ± 7	19 ± 6	0.66	1.34	1.0
Europine (mono, *7S*)	> 300	> 300	34.1	45.5	0.3
Heliotrine (mono, *7S*)	176 ± 31	193 ± 17	4.42	10.4	0.3
Echimidine (open, di, *7R*)	179 ± 17	25 ± 1	7.85	17.3	0.1
Indicine (mono, *7R*)	> 300	210 ± 16	34.2	76.3	0.01
Lycopsamine (mono, *7R*)	> 300	114 ± 18	59.5	73.3	0.01

Cytotoxicity data for PA (except riddelliine) in rat hepatocytes (PH) in primary culture were taken from Gao et al. (2019) and are shown for comparison; Cytotoxicity data show means ± S.D. from n=3 independent experiments; iREP factors are taken from Merz and Schrenk (2016). PAs are grouped according to iREP classes, in alphabetic order

#### Micronuclei formation

The same concentrations tested were also used for the micronuclei assay in HepG2 C9 (CYP3A4) cells. All eleven PAs induced a significant increase in micronuclei counts, with a very different potency, however. With all potent PA congeners, the amount of micronuclei detected increased with increasing concentrations; while at relatively high concentrations, the effect became smaller. This finding is in accordance with the assumption that severe cytotoxicity attenuates micronuclei formation in HepG2 cell cultures exposed to genotoxic PAs. This observation is obviously the basis for the recommendation in the OECD guideline on micronuclei testing (OECD 2016). Following this recommendation, micronuclei counts at concentrations leading to a loss of viability exceeding 50% were, thus, not considered for further modeling. With senecionine (Fig. 1a), micronuclei counts increased at concentrations in the range of 0.1 µM, reached a maximum at about 2.5–5 µM, and declined at 25 µM. In comparison, lycopsamine led to an increase in micronuclei counts at concentrations of 75 µM, rising further at 150 and 300 µM (Fig. 1b).

### Modeling: relative genotoxic potencies

For a calculation of relative genotoxic potencies, we used this kind of data for all PAs tested and modeled them according to the benchmark calculation mode recommended by RIVM and EFSA. This software (PROAST) calculates the best fit of six different algorithms widely used in dose–response modeling. The best four out of these fits are analyzed further and weighted according to their quality of fitting. Figures 2a, b, show examples of the four best fittings obtained for lycopsamine and senecionine. Next, the benchmark concentrations (BMC) leading to a doubling of micronuclei counts over background and their upper and lower 10% confidence limits were calculated. These are given for each congener in Table 1 showing that all PAs tested induced micronuclei with marked differences in the BMCs, their Lower Bounds spanning over more than three, their Upper Bounds over more than two orders of magnitude. The most potent genotoxicants were lasiocarpine and senecionine with all other congeners attributed with an iREP factor of 1.0, except monocrotaline, within the range of 0.01–1.29 μM (LB) and 0.24–2.29 μM (UB). The two *7S*-monoesters europine and heliotrine differed remarkably in their potencies, heliotrine being four- (UB) to eightfold (LB) more potent than europine. Europine was much less potent than an iREP factor of 0.3 would suggest, while the potency of echimidine (iREP factor 0.1) was one order of magnitude lower than for the ‘highly toxic’ congeners. The *7R*-monoesters were the least genotoxic with BMCs between 34.2 and 59.5 (LB) and 73.3 and 76.3 μM (UB) indicating that an iREP factor of 0.01 is also adequate in this test system.

Finally, we wanted to obtain more information on the shape of the concentration–response relationship at the low-effect concentrations. For this purpose, we repeated the micronuclei assays for three selected PAs at these additional concentrations ranges (lasiocarpine: 0.05 and 0.25 µM, retrorsine: 0.01, 0.025 and 0.05 µM, senecionine: 0.01 µM). For retrorsine (Fig. 3a), the fine-tuning for the range of lower concentrations revealed a practical threshold in the range below 1 μM (log = 0) showing no concentration-dependent increase in micronuclei counts. The picture for lasiocarpine (Fig. 3b) was different, i.e., no indication for a practical threshold at a concentration of 0.01 μM was seen. With senecionine (Fig. 3c) concentrations below 0.03 μM exhibited an apparent no-effect range, i.e., no concentration-dependent genotoxicity could be measured. The LB vs. UB intervals (retrorsine: 0.98–1.96 μM; lasiocarpine: 0.01–0.25 μM; senecionine: 0.05–0.2 μM) were quite similar to the ones determined at the higher concentration ranges (Table 1).

## Discussion

Several 1,2-unsaturated PAs have been shown to exert pronounced liver toxicity in humans and animals and cyto- and genotoxicity in mammalian cell culture. It is widely accepted that the cytotoxic effects of PAs are mainly due to the formation of reactive intermediates of the dehydro-retronecine type (Mattocks and White 1971). These exert electrophilic properties and can bind covalently to cellular nucleophilic targets such as glutathione, proteins and nucleic acids (Fu et al. 2004). When structurally different congeners were tested in vitro, a structure–activity relationship was observed with marked differences in the cytotoxic potencies between monoesters on the one hand and certain open-chained and cyclic diesters on the other hand (reviewed in Merz and Schrenk 2016). Such data were obtained, e.g., in bovine kidney epithelial cells (Kim et al. 1993) or HepG2 or HepG2/C3A hepatoblastoma cells (Li et al. 2013; Tamta et al. 2012). In primary cultures of rat hepatocytes, Green et al. (1981) found a marked cytotoxicity with senecionine. Field et al. (2015) used both the LDH and the MTT assays for measuring cytotoxicity of PAs in a chicken hepatocarcinoma cell line in the potency rank order (LDH- or MTT-assay) of lasiocarpine > seneciphylline > senecionine > heliotrine > riddelliine > monocrotaline > riddelliine-N-oxide > intermedine > lycopsamine ≈ lasiocarpine-*N*-oxide ≈ senecionine-*N*-oxide. A combined approach, suggesting iREP factors based on a limited set of date, led to the publication of interim REP factors in an attempt to quantify the relative toxic potency of PA congeners (Merz and Schrenk 2016). In general, this concept was supported by in vitro findings on PA-dependent micronuclei formation (Allemang et al. 2018), DNA adduct formation (Lester et al. 2019) and, genotoxicity-related γH2AX histone phosphorylation (Louisse et al. 2019). Recently, we could identify the cytotoxicity of ten PA congeners belonging to different structural classes in rat hepatocytes in primary culture (Gao et al. 2020). It was found that lasiocarpine and all cyclic diesters tested except monocrotaline, exerted a high cytotoxicity with EC_50_ levels between 4 and 19 μM. Furthermore, the group of monoesters showed a relatively low cytotoxicity with EC_50_ levels above 100 μM, while the open diester (*7R*) echimidine exerted a slightly lower cytotoxicity than the cyclic diesters. When these findings are compared with the data presented here on HepG2 C9 (CYP3A4) cells, it is obvious that the latter are less sensitive than rat hepatocytes towards lasiocarpine and most cyclic diesters. Again, monocrotaline was much less cytotoxic than expected from its structural classification as a cyclic diester. All other open-chained congeners were less cytotoxic with EC_50_ values above 170–300 μM. It remains to be elucidated if the lower sensitivity of human HepG2 C9 (CYP3A4) cells is due to species differences, e.g., in the pattern of metabolism including activation and detoxification pathways. Recently, no marked differences were found when human and rat liver microsomes were used to generate GSH adducts with a number of potent PAs (Geburek et al. 2020). It was shown by Ruan et al. (2014) that a variety of CYP enzymes can activate PAs in human liver microsomes to a variable extent. Thus, it cannot be excluded that the lack of certain CYP enzymes such as CYP2E1 or CYP2B subtypes not expressed in HepG2 C9 cells to a relevant degree may modify the outcome in our system. It needs to be noted, however, that permanent cell lines will always differ to a certain extent from human hepatocytes with respect to CYP expression. Furthermore, these activities are subject to considerable interindividual variability in human hepatocyte or liver microsome preparations as well (Hallifax and Houston 2009).

The possibility was also investigated that a lower level of CYP3A4 expression in HepG2 (CYP3A4) can explain the lower sensitivity in this cell line. BROD activity in HepG2 (CYP3A4) cells was in fact markedly lower than in freshly isolated rat hepatocytes but similar to rat hepatocytes after 24 h in culture. Thus, it appears unlikely that differences in CYP3A4 activity are the only explanation for the higher sensitivity in rat hepatocytes taking into account that an incubation time of 48 h was used in both cell types. Possibly, differences in transmembrane transporters playing a crucial role in the cellular disposition of at least certain PAs (Tu et al. 2013, 2014) may also be relevant.

In vitro genotoxicity of PAs was demonstrated by a number of studies to depend strongly on the congeners tested suggesting a structure–potency relationship. Xia et al. (2008) incubated PAs with rat liver microsomes in the presence of calf thymus DNA and found the following rank order of DNA adduct formation: retrorsine > retrorsine-*N*-oxide > heliotrine. Wang et al. (2005) could find identical DNA adducts formed in in vitro incubations of retrorsine with liver microsomes and in the liver of retrorsine-treated rats. Other PAs shown for their capacity to exert DNA binding in vivo were senecionine, seneciphylline (Eastman et al. 1982), retrorsine, lasiocarpine, and lycopsamine (Xia et al. 2013). Although 1,2-unsaturated PAs are widely accepted as being genotoxic in mammalian test systems, the outcome of bacterial mutagenicity tests, in particular the Ames test, is equivocal (reviewed in Merz and Schrenk 2016). A number of authors reported very minor or no mutagenic effects in bacteria treated with various PA congeners which are in agreement with our findings. Any mutagenicity of PAs in bacteria is likely to depend on external metabolic activation by S9-mix. Active metabolites then need to enter the bacterial cell to exert mutagenicity. It is unclear if the reactivity, i.e., the chemical half-life of these metabolites allows a mutagenic effect keeping in mind that active uptake mechanisms seem to be important for PA toxicity in mammalian cells (Tu et al. 2013, 2014). It remains questionable if similar or homologous transporters exist in *Salmonella*. The fact that several PAs exerted bactericidal activity in the presence of S9-mix may indicate a possible attack of reactive metabolites at the outer bacterial wall and/or membrane resulting in cell death.

With all PAs tested, a positive response was obtained in the micronucleus assay, although substantial differences in relative potencies were obtained. It was evident that micronuclei counts increased at much lower concentrations that cytotoxicity. Both outcomes although probably depending on an electrophilic attack via the same/similar reactive intermediate/s differed in their concentrations–response characteristics. This finding is not surprising keeping in mind the different types of targets and the different assays. Similar findings were reported by Allemang et al. (2018) who found significant increases in micronuclei counts in HepaRG cells at PA concentrations which did not affect relative cell survival. Higher PA concentrations were found to lead to less pronounced increases in micronuclei. Similar results were reported in other micronuclei studies (Dorn et al. 2008, Schuler et al. 2010) and are probably due to marked cytotoxicity.

Numerous PAs turned out to be potent mutagens in *Drosophila*, bacteria and mammalian cells in culture. In Drosophila, Clark et al. (1960) identified heliotrine and lasiocarpine as ‘potent’, and senecionine, echimidine and echinatine as ‘moderate’ mutagens. *N*-oxides exerted attenuated effects in comparison to the ‘parent’ congener PAs. Later, Cook and Holt (1966) using the same assay, classified retrorsine as ‘moderate’, while retrorsine-*N*-oxide was markedly less active. Frei et al. (1992) investigated the genotoxic potency of 16 PAs using the ‘wing spot test’ in *Drosophila*. The relative potencies in percent were: Senkirkine 100, monocrotaline 90.0, seneciphylline 54.5, senecionine 39.1, heliotrine 13.4, retrorsine 8.3, symphytine 3.8, intermedine 0.49, indicine 0.27, and lycopsamine 0.19. With the exception of heliotrine (*7S*-configuration of the necine base), this test system confirmed a rank order of genotoxicity of cyclic diesters > open-chained diesters > monoesters.

Genotoxic effects reported for 1,2-unsaturated PAs comprise increased UDS formation in cultured rat hepatocytes with lasiocarpine and monocrotaline (Williams et al. 1980), in rat, mouse and hamster hepatocytes with monocrotaline, petasitenine, senkirkine, senecionine, and seneciphylline (Mori et al. 1985), and in rat and human hepatocytes with senkirkine (Schehrer et al. 2000). Chromosomal alterations including induction of micronuclei, chromosomal aberrations and sister chromatid exchange were also reported for a number of PAs. Retrorsine led to the formation of micronuclei in human lymphocytes incubated with rat liver S9-mix and in the human hepatoma cell line HepG2 (Kevekordes et al. 2001). In rat bone marrow (Proudlock et al. 1997) and in mouse bone marrow and fetal liver (Sanderson and Clark 1993), monocrotaline led to a significant increase in micronuclei, while a study with riddelliine in mice was negative (Mirsalis et al. 1993). In vitro studies in rat hepatocyte culture revealed enhanced micronuclei formation with retrorsine and monocrotaline with active concentrations of 1–10 μM for retrorsine and 3–30 μM for monocrotaline (Müller-Tegethoff et al. 1997). In a recent study by Allemang et al. (2018), eleven PA congeners and four PA *N*-oxides were analyzed for their potency to induce micronuclei in human HepaRG cells. Eight congeners were identical to those tested in the present study, i.e., echimidine, europine, heliotrine, indicine, lasiocarpine, lycopsamine, monocrotaline, and retrorsine. The authors treated HepaRG cells over 24 h with increasing concentrations of PAs and analyzed micronuclei levels using flow cytometry. In the present study, HepG2 C9 cells were treated over 72 h and micronuclei were counted via microscopical inspection. The latter method allows the unequivocal identification of micronuclei resulting in very low counts in untreated cells. Interestingly, the data reported by Allemang et al. are in excellent agreement with our findings for the eight congeners analyzed in both studies. For most congeners, however, the ranges (Upper bounds–Lower bounds) were somewhat lower in our study. This may be due to the longer incubation period, the lower background counts and a possible higher sensitivity of HepG2 C9 cells. In agreement with our findings, monocrotaline was much less potent than a RPF of 1.0 would indicate, i.e., in in vitro studies on DNA adduct formation (Lester et al. 2019) or gentotoxicity (Allemang et al. 2018, Louisse et al. 2019). Furthermore, europine seems to be over-estimated by the iREP factor of 0.3, at least when in vitro findings from our study and studies by others (Allemang et al. 2018, Lester et al. 2019, Louisse et al. 2019) are considered.

To obtain more detailed information on the shape of the concentration–response curves of genotoxicity at low concentrations, we fine-tuned the concentration ranges for retrorsine, lasiocarpine, and senecionine. It could be shown that the concentration–response curves for retrorsine and senecionine are hypolinear exerting a practical no-effect threshold. In this range below 0.03 (senecionine) or 1.0 μM, no increase in micronuclei counts was found. These findings indicate that a linear extrapolation of the rates of genotoxicity events from high to low dose, and probably also for suspected cancer incidences over-estimates the risk. It was interesting to find that for the most toxic congener, lasiocarpine, this assumption could not be made supporting the conclusion that considerable qualitative and quantitative differences exist between individual congeners.

Lester et al. (2019) measured PA kinetics and DNA adduct formation in rat sandwich culture hepatocytes. Linearity of the dose response of DNA adduct formation was examined for a few PAs at higher concentrations than used in the micronuclei experiments here, but comparison of the different events across dose and time seems to be pointing to the importance of cellular defence mechanisms like DNA repair at very low concentrations.

Taken together, our findings clearly demonstrate that all eleven 1,2-unsaturated PAs are genotoxic in a metabolically competent human cell model. The lack of mutagenicity in *Salmonella typhimurium* strains seems to be due to certain restrictions of the method such as the need for metabolic activation outside the bacterial cell. The potency for micronuclei induction as a hallmark of genotoxicity in mammalian cells clearly depended on the type of PA. The open *7S*-configurated diester lasiocarpine and all cyclic diesters except monocrotaline were very potent genotoxicants, lasiocarpine being effective at very low concentrations, i.e., at 0.01–0.49 μM. Thus, a common iREP factor of 1.0 for these congeners can be confirmed. The much lower geno- and cytotoxicity of monocrotaline may be due to special properties in the uptake (Chen et al. 2019) and metabolism of this congener. The fact that monocrotaline also exerted a low cytotoxicity in rat PH suggests that the lack of CYP2B enzymes in HepG2 C9 cells is unlikely, however, to explain the low toxicity in these cells since rat PH express CYP2B. Furthermore, the very limited database for the derivation of iREP factors may explain why a factor of 1.0 for monocrotaline is not confirmed in more refined experimental models. Obviously, the relative toxicity of monocrotaline is markedly over-estimated by this factor. Another congener probably being over-estimated in the iREP concept according to our results is europine. For echimidine and heliotrine an intermediate potency seems to be warranted while the *7R*-configurated monoesters were confirmed to be of low cyto- and genotoxicity. Our findings suggest that additional studies are needed to refine the concept of REP factors but confirm the notion that the assumption of equal genotoxicity of PAs, a hallmark of the carcinogenic process, cannot be supported from a scientific perspective and is a pronounced overestimation of the risk. It is, thus, recommended to establish a more robust system of relative potencies defining, e.g., groups of congeners with high, intermediate and low genotoxic potency.

## Electronic supplementary material

Below is the link to the electronic supplementary material.

Supplementary material 1 (DOCX 31 kb)

Supplementary material 2 (DOCX 31 kb)

## Abbreviations

BMC: Benchmark concentration
BROD: 7-Benzoxyresorufin-*O*-dealkylase
CYP: Cytochrome P450
DAPI: 4′,6-diamidino-2-phenylindole
DMEM: Dulbecco’s modified Eagle’s Medium
DMSO: Dimethyl sulfoxide
DSMZ: German collection of microorganisms and cell cultures GmbH
EC_50_: Effective concentration 50%
EDTA: Ethylene diamine tetraacetate
EFSA: European Food Safety Authority
FAU: Formazine attenuation unit
FCS: Fetal calf serum
GSH: Glutathione
γH2AX: Gamma-histone H2AX
HG: High glucose
HVOC: Hepato-venoocclusive disease
HCl: Hydrogen chloride
iREP: Interim relative potency
KCl: Potassium chloride
LB: Lower bound
LC/MS/MS: Liquid chromatography/mass spectrometry/mass spectrometry
LDH: Lactate dehydrogenase
LOD: Limit of detection
MgCl_2_: Magnesium chloride
MMS: Methyl methane sulfonate
MTT: 3-(4,5-dimethylthiazol-2-yl)-2,5-diphenyltetrazolium bromide
NH_4_OH: Ammonium hydroxide
NMR: Nuclear magnetic resonance
OECD: Organisation for Economic Co-operation and Development
PA: Pyrrolizidine alkaloid
PH: Primary hepatocytes
PBS: Phosphate-buffered saline
RIVM: Rijksinstituut voor Volksgezondheid en Milieu
S9 mix: Supernatant mixture obtained at 9,000 g
UB: Upper bound
UDS: Unscheduled DNA synthesis
