# Supplementary material for: Structure-dependent genotoxic potencies of selected pyrrolizidine alkaloids in metabolically competent HepG2 cells
Source: Arch Toxicol. 2020 Sep 10;94(12):4159–72. doi: 10.1007/s00204-020-02895-z (PMC7655576; doi:10.1007/s00204-020-02895-z)
Supplement: Supplementary file 1 — Supplementary material 1 (DOCX 31 kb) [file 204_2020_2895_MOESM1_ESM.docx]

**Table S1.** Fluctuation Ames test in *S. typhimurium* TA98. The data represent means and standard deviations (S.D.) from n= 3 independent experiments.

| Compound | Concentration [µM] | - S9-mix  positive wells [%] | S. D. [%] | + S9-mix  positive wells [%] | S. D. [%] |
| --- | --- | --- | --- | --- | --- |
| echimidine | 1 | 6.94 | 2.60 | 2.08 | 1.70 |
|  | 5 | 4.17 | 1.70 | 1.39 | 0.98 |
|  | 25 | 5.56 | 3.93 | 1.39 | 1.96 |
|  | 75 | 2.08 | 2.95 | 2.78 | 0.98 |
|  | 150 | 0.69 | 0.98 | 2.78 | 1.96 |
|  | 300 | 6.25 | 3.40 | 4.17 | 3.40 |
| europine | 1 | 2.78 | 2.60 | 2.78 | 0.98 |
|  | 5 | 5.56 | 0.98 | 4.17 | 1.70 |
|  | 25 | 7.64 | 0.98 | 0.69 | 0.98 |
|  | 75 | 4.86 | 3.54 | 1.39 | 1.96 |
|  | 150 | 1.39 | 1.96 | 2.78 | 0.98 |
|  | 300 | 5.56 | 3.54 | 3.47 | 0.98 |
| heliotrine | 1 | 9.72 | 5.20 | 2.78 | 2.08 |
|  | 5 | 4.17 | 2.95 | 3.47 | 2.71 |
|  | 25 | 5.56 | 0.98 | 2.08 | 1.73 |
|  | 75 | 2.08 | 2.95 | 3.47 | 2.71 |
|  | 150 | 4.86 | 1.96 | 4.17 | 3.45 |
|  | 300 | 2.78 | 0.98 | 4.17 | 2.33 |
| indicine | 1 | 2.08 | 2.77 | 2.77 | 3.18 |
|  | 5 | 0.69 | 0.69 | 0.69 | 1.20 |
|  | 25 | 2.08 | 2.08 | 2.08 | 2.08 |
|  | 75 | 0.69 | 0.69 | 0.69 | 1.20 |
|  | 150 | 0.69 | 1.38 | 1.38 | 2.40 |
|  | 300 | 2.77 | 0.69 | 0.69 | 1.20 |
| lasiocarpine | 1 | 2.08 | 2.08 | 0.00 | 0.00 |
|  | 5 | 0.69 | 1.20 | 1.38 | 1.20 |
|  | 25 | 2.77 | 2.40 | 3.47 | 1.20 |
|  | 75 | 1.38 | 1.20 | 2.08 | 0.00 |
|  | 150 | 0.00 | 0.00 | 0.69 | 1.20 |
|  | 300 | 1.38 | 2.40 | 2.08 | 2.08 |
| lycopsamine | 1 | 2.08 | 3.60 | 1.38 | 1.20 |
|  | 5 | 2.08 | 0.00 | 0.00 | 0.00 |
|  | 25 | 2.08 | 2.08 | 0.69 | 1.20 |
|  | 75 | 1.38 | 1.20 | 1.38 | 1.20 |
|  | 150 | 0.00 | 0.00 | 0.69 | 1.20 |
|  | 300 | 0.00 | 0.00 | 1.38 | 1.20 |
| monocrotaline | 1 | 6.25 | 4.50 | 4.17 | 2.95 |
|  | 5 | 4.17 | 4.50 | 5.56 | 3.54 |
|  | 25 | 1.39 | 0.98 | 2.08 | 1.70 |
|  | 75 | 1.39 | 0.98 | 2.78 | 2.60 |
|  | 150 | 1.39 | 0.98 | 2.08 | 2.95 |
|  | 300 | 2.08 | 1.70 | 2.78 | 2.60 |
| retrorsine | 1 | 0.00 | 0.00 | 2.08 | 2.08 |
|  | 5 | 3.47 | 1.20 | 2.08 | 2.08 |
|  | 25 | 1.38 | 2.40 | 0.00 | 0.00 |
|  | 75 | 1.38 | 2.40 | 1.38 | 2.40 |
|  | 150 | 2.08 | 2.08 | 0.69 | 1.20 |
|  | 300 | 2.77 | 3.18 | 0.69 | 1.20 |
| riddelliine | 1 | 2.08 | 1.70 | 1.39 | 0.98 |
|  | 5 | 3.47 | 0.98 | 2.08 | 1.70 |
|  | 25 | 4.17 | 1.70 | 3.47 | 0.98 |
|  | 75 | 3.47 | 2.60 | 2.08 | 1.70 |
|  | 150 | 3.47 | 0.98 | 3.47 | 0.98 |
|  | 300 | 7.64 | 2.60 | 2.78 | 0.98 |
| senecionine | 1 | 1.38 | 1.20 | 0.69 | 1.20 |
|  | 5 | 3.47 | 3.18 | 1.38 | 2.40 |
|  | 25 | 2.77 | 1.20 | 1.38 | 1.20 |
|  | 75 | 0.69 | 1.20 | 2.08 | 0.00 |
|  | 150 | 2.08 | 0.00 | 2.77 | 1.20 |
|  | 300 | 0.00 | 0.00 | 2.77 | 2.40 |
| seneciphylline | 1 | 2.78 | 3.93 | 1.39 | 0.98 |
|  | 5 | 0.00 | 0.00 | 2.08 | 1.70 |
|  | 25 | 2.08 | 1.70 | 0.69 | 0.98 |
|  | 75 | 0.69 | 0.98 | 0.69 | 0.98 |
|  | 150 | 2.78 | 2.60 | 2.78 | 1.96 |
|  | 300 | 2.78 | 2.60 | 0.69 | 0.98 |
