# Supplementary material for: Structure-dependent genotoxic potencies of selected pyrrolizidine alkaloids in metabolically competent HepG2 cells
Source: Arch Toxicol. 2020 Sep 10;94(12):4159–72. doi: 10.1007/s00204-020-02895-z (PMC7655576; doi:10.1007/s00204-020-02895-z)
Supplement: Supplementary file 2 — Supplementary material 2 (DOCX 31 kb) [file 204_2020_2895_MOESM2_ESM.docx]

**Table S2.** Fluctuation Ames test in *S. typhimurium* TA100. The data represent means and standard deviations (S.D.) from n= 3 independent experiments.

| Compound | Concentration [µM] | - S9-mix  positive wells [%] | S. D. [%] | + S9-mix  positive wells [%] | S. D. [%] |
| --- | --- | --- | --- | --- | --- |
| echimidine | 1 | 9.03 | 2.60 | 3.47 | 0.98 |
|  | 5 | 6.25 | 3.40 | 9.03 | 2.60 |
|  | 25 | 9.03 | 0.98 | 2.78 | 0.98 |
|  | 75 | 7.64 | 5.20 | 5.56 | 1.96 |
|  | 150 | 7.64 | 2.60 | 6.25 | 1.70 |
|  | 300 | 6.94 | 2.60 | 4.86 | 2.60 |
| europine | 1 | 8.33 | 3.40 | 9.03 | 2.60 |
|  | 5 | 6.94 | 3.54 | 7.64 | 0.98 |
|  | 25 | 10.42 | 1.70 | 4.86 | 2.60 |
|  | 75 | 15.28 | 0.98 | 6.25 | 1.70 |
|  | 150 | 13.19 | 3.54 | 6.25 | 1.70 |
|  | 300 | 10.42 | 3.40 | 4.17 | 1.70 |
| heliotrine | 1 | 5.56 | 3.54 | 3.47 | 0.98 |
|  | 5 | 9.03 | 3.54 | 5.56 | 1.96 |
|  | 25 | 4.86 | 2.60 | 6.94 | 4.91 |
|  | 75 | 8.33 | 4.50 | 3.47 | 1.96 |
|  | 150 | 4.17 | 3.40 | 5.56 | 1.96 |
|  | 300 | 11.11 | 1.96 | 7.64 | 3.93 |
| indicine | 1 | 2.08 | 2.08 | 2.77 | 3.18 |
|  | 5 | 2.77 | 3.18 | 1.38 | 2.40 |
|  | 25 | 3.47 | 2.40 | 1.38 | 2.40 |
|  | 75 | 2.08 | 3.60 | 1.38 | 2.40 |
|  | 150 | 1.38 | 1.20 | 3.47 | 2.40 |
|  | 300 | 4.16 | 3.60 | 6.25 | 2.08 |
| lasiocarpine | 1 | 2.08 | 2.08 | 2.60 | 1.04 |
|  | 5 | 2.77 | 1.20 | 3.12 | 2.68 |
|  | 25 | 4.86 | 3.18 | 6.25 | 2.94 |
|  | 75 | 2.08 | 2.08 | 6.77 | 3.12 |
|  | 150 | 3.47 | 1.20 | 7.29 | 3.60 |
|  | 300 | 4.16 | 4.16 | 6.77 | 4.61 |
| lycopsamine | 1 | 4.16 | 2.08 | 4.86 | 3.18 |
|  | 5 | 2.77 | 2.40 | 0.69 | 1.20 |
|  | 25 | 4.86 | 3.18 | 3.47 | 4.33 |
|  | 75 | 4.86 | 4.81 | 1.38 | 1.20 |
|  | 150 | 0.69 | 1.20 | 3.47 | 4.33 |
|  | 300 | 4.16 | 2.08 | 4.16 | 3.60 |
| monocrotaline | 1 | 6.94 | 1.96 | 8.33 | 2.95 |
|  | 5 | 7.64 | 0.98 | 6.94 | 3.54 |
|  | 25 | 6.94 | 5.47 | 7.64 | 3.93 |
|  | 75 | 10.42 | 1.70 | 3.47 | 0.98 |
|  | 150 | 12.50 | 1.70 | 2.78 | 0.98 |
|  | 300 | 9.03 | 2.60 | 4.17 | 1.70 |
| retrorsine | 1 | 3.47 | 1.20 | 1.56 | 1.99 |
|  | 5 | 5.55 | 2.40 | 3.64 | 2.62 |
|  | 25 | 5.55 | 4.33 | 5.72 | 3.55 |
|  | 75 | 3.47 | 4.33 | 5.72 | 3.55 |
|  | 150 | 6.25 | 2.08 | 4.16 | 3.80 |
|  | 300 | 4.16 | 2.08 | 6.77 | 1.99 |
| riddelliine | 1 | 9.03 | 2.60 | 2.78 | 1.96 |
|  | 5 | 6.94 | 0.98 | 2.08 | 1.70 |
|  | 25 | 10.42 | 1.70 | 2.08 | 1.70 |
|  | 75 | 11.81 | 5.47 | 2.78 | 0.98 |
|  | 150 | 11.81 | 3.54 | 6.94 | 0.98 |
|  | 300 | 11.11 | 2.60 | 6.94 | 0.98 |
| senecionine | 1 | 6.25 | 3.60 | 2.77 | 1.20 |
|  | 5 | 6.25 | 5.51 | 4.16 | 2.08 |
|  | 25 | 4.86 | 4.81 | 6.25 | 3.60 |
|  | 75 | 5.55 | 1.20 | 6.25 | 7.51 |
|  | 150 | 4.16 | 2.08 | 6.94 | 5.24 |
|  | 300 | 5.55 | 1.20 | 6.94 | 6.01 |
| seneciphylline | 1 | 4.17 | 2.95 | 7.64 | 6.87 |
|  | 5 | 4.86 | 1.96 | 7.64 | 5.20 |
|  | 25 | 1.39 | 1.96 | 6.25 | 3.40 |
|  | 75 | 10.42 | 4.50 | 7.64 | 6.44 |
|  | 150 | 9.03 | 5.47 | 7.64 | 2.60 |
|  | 300 | 5.56 | 3.54 | 6.25 | 4.50 |
